# Dry‐salted cod (*Gadus morhua*) rehydration assisted by pulsed electric fields: modelling of mass transfer kinetics

**DOI:** 10.1002/jsfa.11852

**Published:** 2022-03-15

**Authors:** Jessica Genovese, Silvia Tappi, Urszula Tylewicz, Fabio D'Elia, Ana C. De Aguiar Saldanha Pinheiro, Pietro Rocculi

**Affiliations:** ^1^ Department of Agricultural and Food Sciences, Alma Mater Studiorum University of Bologna Cesena Italy; ^2^ Interdepartmental Centre for Agri‐Food Industrial Research, Alma Mater Studiorum University of Bologna Cesena Italy

**Keywords:** pulsed electric field, rehydration kinetics, mass transfer, Peleg model, dry‐salted cod

## Abstract

**BACKGROUND:**

Dry‐salted cod (*Gadus morhua*) must be rehydrated before consumption and this step can take up to 5 days. Desalting of cod on an industrial scale poses many problems, mainly related to the long processing times and the quality of the final product. For this reason, many researchers have focused on finding new desalting methods to improve mass transfer. The application of pulsed electric fields (PEF) has been proposed as an alternative method for improving mass transfer in many food processes. However, there is no previous literature on the use of PEF to improve animal tissue rehydration. Therefore, the present study aimed to investigate the influence of two PEF pre‐treatments [PEF (1) 500 V cm^−1^ and PEF (2) 1000 V cm^−1^] on mass transport kinetics during the rehydration process of salted cod. The rehydration process was carried out under static conditions for 6 days, immersing dry‐salted cod samples in tap water (5 ± 0.5 °C).

**RESULTS:**

The results show that the use of PEF technology increases the rate of the rehydration process of dry‐salted cod and influences the redistribution of salt. In general, the samples pre‐treated with PEF showed higher weight gain and lower salt loss than the control samples during the rehydration process.

**CONCLUSION:**

The application of PEF prior to rehydration of salted cod samples could be of interest to the food industry as a result of a higher process yield (higher weight gain) and the possibility to reduce the water renewal because less NaCl is lost in the wastewater. © 2022 The Authors. *Journal of The Science of Food and Agriculture* published by John Wiley & Sons Ltd on behalf of Society of Chemical Industry.

## INTRODUCTION

Dry‐salted cod (*Gadus morhua*) is a highly prized product traditionally imported from Mediterranean countries and marketed with varying moisture content depending on the extent of the dehydration process. In the traditional production of salted cod, alternate layers of product and dry salt crystals are stored in stacked piles, where the product is kept for approximately 2–8 weeks, depending on the desired degree of dehydration.^1^ Generally, the final product reaches a salt concentration of 200–250 g kg^−1^ and a final water content of 500–650 g kg ^−1^. In addition, the reduction in water activity as a result of the removal of water and the increase in salt content in the tissues leads to the inhibition of spoilage processes, thus increasing the shelf‐life and the stability of the fish.^2^ Before consumption, the fish must be rehydrated/desalted to reduce the salt concentration to a level suitable for consumption (in the range 20–30 g kg^−1^). This final step is usually carried out by the consumer by immersing the product in stagnant water at room temperature or refrigerated conditions.^3^ At this stage, a two‐ways mass transfer takes place, resulting in the leaching of sodium chloride ions from the tissue matrix and, in contrast, in the uptake of water, with a tendency to increase cod weight and volume and to redissolve salt and proteins. Many factors can affect the kinetics of mass transfer in drying and desalting operations, including the quality and chemical composition of the raw material and the method used for salting. Salting leads to significant changes in the composition and structure of the tissue, which in turn affects the behaviour during dehydration and rehydration.^1^, ^4^

Considering that market trends are moving towards ready‐to‐use products, the cod industry is adapting its processes to consumer requirements and, currently, the desalting step is frequently carried out and included in industrial operations. However, the rehydration of cod on an industrial‐scale is associated with numerous problems, mainly related to the long processing times (generally around 2–3 days, depending on the thickness of the fish pieces) and the quality of the final product. Traditional large‐scale rehydration is similar to the desalting process performed by consumers at home.^3^, ^5^ It employs the handling of large volumes of water because several water renewals are required to improve the efficiency of the process (also in terms of yield). Therefore, industrial desalting is also critical in terms of wastewater management. The latter should be considered as a process parameter for optimising the desalting process.^6^ Indeed, the residual brine is an environmentally harmful wastewater characterised by dissolved and suspended solids (mainly Na^+^ and Cl^−^ ions, and, to a lesser extent, dissolved proteins) and must be treated before discharge into the municipal sewage system.^7^ For this reason, industrial desalting of cod should be optimised to produce a commercial product with higher yield (high weight ratio between desalted cod/initial salted cod) and right salt concentration and nutritional value, at the same time as minimising the amount of waste. In recent years, many researchers have focused on finding new methods to improve the kinetics of desalting/rehydration processes, such as the use of vacuum pulses,^8^ high pressure^9^ or high‐intensity ultrasound.^10^

The application of pulsed electric fields (PEF) has been proposed as an alternative method to improve mass transfer in many food processes. PEF technology consists of electrical treatment of short duration (from several nanoseconds to several milliseconds) with electric field strengths ranging from 100–300 V cm^−1^ to 20–80 kV cm^−1^.^11^ High electric fields (> 20 kV cm^−1^) are usually used to inactivate spoilage and pathogenic microorganisms and quality‐relevant enzymes.^12^ Lower electric field strengths (0.5–3 kV cm^−1^) are used alone or in combination with other processes in the food industry and could lead to temporary or permanent loss of cell membrane semi‐permeability.^13^ Cell membranes can be considered as a physical barrier to diffusion processes, such that the degree of their permeabilisation can influence mass transfer phenomena.

To the best of our knowledge, there is no previous literature on the use of PEF treatments to improve the desalting of foods. For this reason, the present study focused on testing the influence of PEF pre‐treatment of salted cod on the kinetics of mass transfer during desalting/rehydration process.

## MATERIALS AND METHODS

### Raw materials

Dry‐salted cod (*G. morhua*) fillets were supplied by a local importer (Tagliapietra e figli s.r.l, Venice, Italy) and, prior to desalting experiments, they were manually cut in cubic‐shape pieces (2 × 2 × 2 cm), obtained from the upper part of the fillet. They had an average weight of 13.8 ± 1.1 g, an initial moisture content of 576.7 ± 5.4 g kg^−1^, and a fat and protein content of 1 and 160 g kg^−1^, respectively. The fillets were kept refrigerated at 4 ± 1 °C before trials.

### PEF treatments

PEF treatments were performed using a lab‐scale PEF unit delivering a maximum output voltage and current of 8000 V and 60 A, respectively (Mod. S‐P7500; Alintel, Pieve di Cento, Italy). The generator provides monopolar near‐rectangular pulses and adjustable pulse duration (5–20 μs), pulse frequency (50–500 Hz) and total treatment time (1–600 s). The treatment chamber (length 50 mm, width 50 mm, height 50 mm) consisted of two parallel stainless‐steel electrodes (thickness 3 mm) with a 47‐mm fixed gap. Output voltage and current were monitored using a PC‐oscilloscope (Picoscope 2204a; Pico Technology, Saint Neots, UK). Samples were treated at room temperature in tap water, with an initial electrical conductivity of 396 ± 5 μs cm^−1^ at 25 °C (EC‐meter Mod. Basic 30; Crison, Barcelona, Spain). Trials were conducted filling the treatment chamber with a product‐to‐water ratio of around 1:5 (w/w) and delivering *n* = 1000 pulses at fixed amplitude (10 ± 1 μs) and frequency (100 Hz). Treatments were performed by applying two different electric field strengths/specific energy inputs (500 V cm^−1^ and 1000 V cm^−1^, 6.8 ± 1.3 kJ kg^−1^ and 23.1 ± 2.4 kJ kg^−1^, respectively) chosen on the basis of preliminary experimental trials. Temperature changes as a result of PEF treatments were negligible. The cod samples were named control (untreated), and PEF (1) and PEF (2) (pre‐treated).

### Rehydration experiments

The rehydration process was carried out in static conditions immersing dry‐salted cod samples in cold tap water (5 ± 0.5 °C) and using a cod‐to‐water ratio of 1:10 (w/w). For the investigation of rehydration kinetics, total weight gain, moisture content, salt (NaCl) content and water activity were determined at 0, 4, 6, 24, 48, 72, 96, 120 and 144 h of the rehydration process.

### Rehydration kinetics

The rehydration of dry‐salted cod implies two mass transports because samples both gain water and lose salt. In the present study, the rehydration kinetics were investigated considering the global evolution of the net sample weight taken at regular time intervals. For each sampling point five cod samples were drained from surface water with absorbent paper and weighed. Therefore, relative mass changes ($∆M_{t}^{o}$), water uptake ($∆M_{t}^{w}$), solute loss ($∆M_{t}^{s}$), and salt loss ($∆M_{t}^{\text{NaCl}}$) were estimated according to:

(1) $$∆M_{t}^{o}=\left(M_{t}^{o}-M_{0}^{o}\right)/M_{0}^{o}$$

(2) $$∆M_{t}^{w}=\left(M_{t}^{o}∙x_{t}^{w}-M_{0}^{o}∙x_{0}^{w}\right)/M_{0}^{o}$$

(3) $$∆M_{t}^{S}=\left(M_{t}^{o}∙x_{t}^{S}-M_{0}^{o}∙x_{0}^{S}\right)/M_{0}^{o}$$

(4) $$∆M_{t}^{\text{NaCl}}=\left(M_{t}^{o}∙x_{t}^{\text{NaCl}}-M_{0}^{o}∙x_{0}^{\text{NaCl}}\right)/M_{0}^{o}$$

where $M_{t}^{o}$ and $M_{0}^{o}$ are the cod weight (g) at the sampling time t and 0. $x_{t}^{w}$ and $x_{0}^{w}$ the cod water weight fractions. $x_{t}^{s}$ and $x_{0}^{s}$ the cod solutes weight fractions. $x_{t}^{\text{NaCl}}$ and $x_{0}^{\text{NaCl}}$ the cod NaCl weight fractions at time t and 0, respectively.

### Kinetic model

To describe the rehydration kinetics of dry‐salted cod, Peleg's empirical model^14^ was considered:

(5) $$M_{t}^{w}-M_{0}^{w}=\frac{1}{k_{1}^{w}+k_{2}^{w}∙t}$$

(6) $$M_{t}^{S}-M_{0}^{S}=-\frac{1}{k_{1}^{S}+k_{2}^{S}∙t}$$

(7) $$M_{t}^{\text{NaCl}}-M_{0}^{\text{NaCl}}=-\frac{1}{k_{1}^{\text{NaCl}}+k_{2}^{\text{NaCl}}∙t}$$

where *k*_1_ is a kinetic parameter (Peleg rate constant) and *k*_2_ is related to equilibrium moisture content (Peleg capacity constant).

In the present study, the same equation, rewritten as:

(8) $$M_{t}^{o}-M_{0}^{o}=\frac{1}{k_{1}^{o}+k_{2}^{o}∙t}$$

was also used to model total mass change kinetics:^15^

This kinetic model offers the advantage that calculating $\frac{1}{k1}$ and $\frac{1}{k2}$ is possible to obtain the initial rate value of mass transfer parameters and the one at the equilibrium condition.

### Analytical determinations

The cod moisture content was determined gravimetrically by oven drying at 105 ± 1 °C until a constant weight was achieved. Sodium chloride was determined after sample homogenisation in distilled water using an Ultra‐Turrax (IKA, Staufen, Germany) at 11200 × *g* for 1 min and centrifugation to remove debris left in the sample. The chloride ion concentration was determined by titration of the centrifuged sample with standard AgNO_3_ solution (0.1 mol L^−1^) and K_2_CrO_4_ as indicator (Mohr's method).^16^ Water activity (*a*_*w*_) was measured at 25 °C using a dew point water activity meter with ±0.003 a_w_ accuracy (Aqua Lab. Decagon Devices, Pullman, WA, USA). For each analytical determination, five cod samples, randomly distributed among two experimental groups (Control and PEF‐treated), were used at each sampling time.

### Statistical analysis

Significant differences between results were calculated by parametric analysis of variance and Tukey's multiple comparison. *P* < 0.05 was considered statistically significant. If a Shapiro–Wilk test for normality and Levene's test for homoscedasticity of data resulted in statistical significance (*P* < 0.05), a non‐parametric multiple range test Kruskal–Wallis and Holm stepwise adjustment were used with a significance level of 95% (*P* < 0.05) (R Foundation for Statistical Computing. Vienna, Austria). All results were expressed as the mean ± SD of replications (*n* = 5 replications per PEF treatment and time steps of the rehydration process). To estimate the kinetic model constants (*k*_1_, *k*_2_), SE and the coefficient of determination (*R* ^2^), non‐linear regression was carried out by means of the quasi‐Newton calculus algorithm using Statistica, version 6.0 (TIBCO, Palo Alto, CA, USA).

## RESULTS

**Figure 1:**
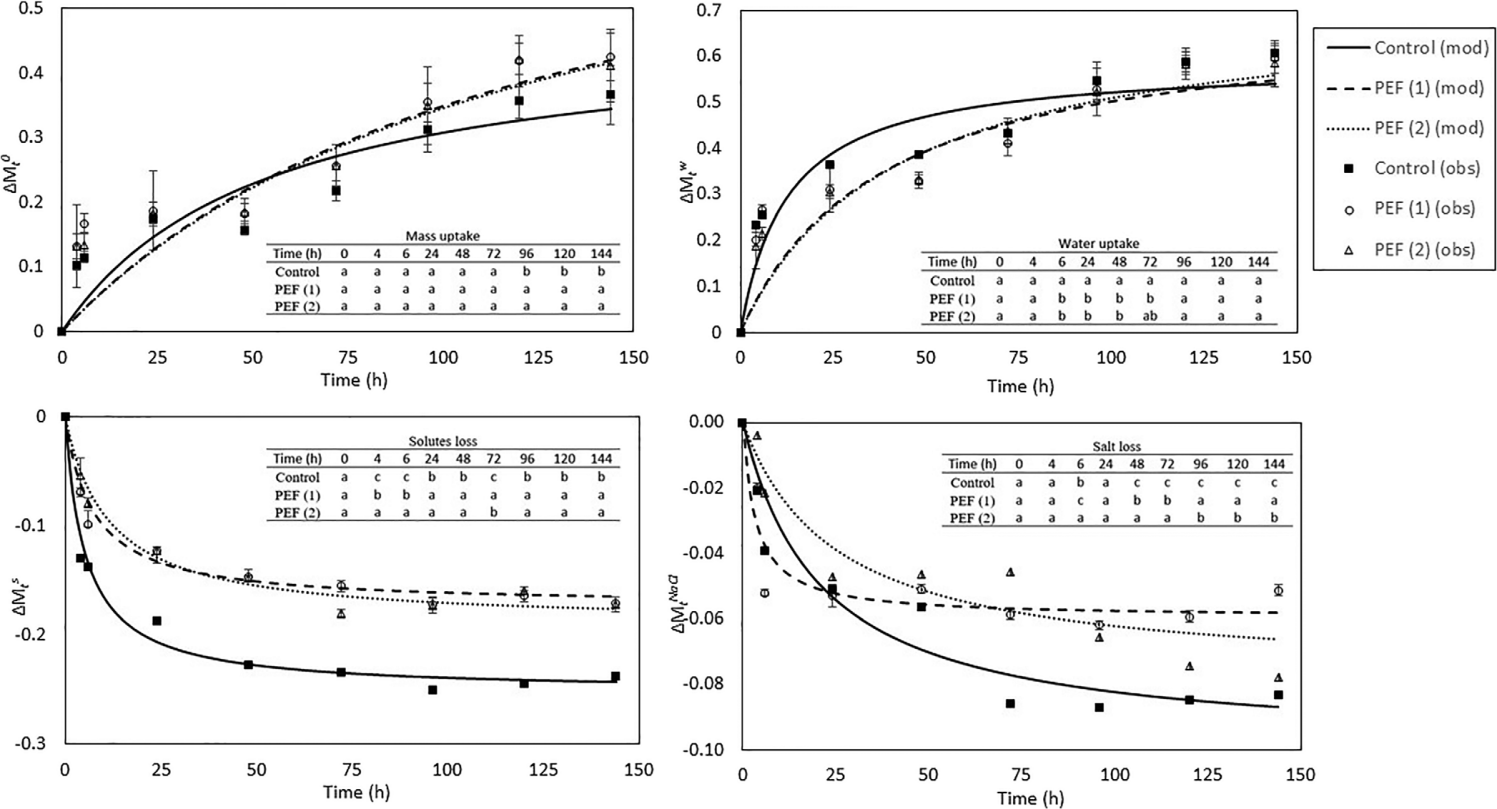
Mass uptake ($∆M_{t}^{o}$), water uptake ($∆M_{t}^{w}$), solute loss $∆M_{t}^{S}$) and salt loss ($∆M_{t}^{\text{NaCl}}$) of untreated and PEF pre‐treated dry‐salted cod samples as a function of the rehydration time. Experimental data (obs) with the curve fitting (mod) are shown. Error bars correspond to the SD of *n* = 5. Different letters on the same column correspond to significantly differences (*P* < 0.05) between samples.

The kinetics of mass $\left(∆M_{t}^{o}\right)$, water uptake $\left(∆M_{t}^{w}\right)$, solutes $\left({∆M}_{t}^{S}\right)$ and salt loss $\left(∆M_{t}^{\text{NaCl}}\right)$ during rehydration of dry‐salted cod, modelled according to Peleg's equation, are shown in Fig. 1. The constants of Peleg's equation (*k*_1_ and *k*_2_) and their reciprocal values are listed in Table 1. Comparing the observed and calculated values for the mass fraction and considering the *r* ^2^ values (between 0.656 and 0.955), the model confirms that it efficiently describes the kinetics of mass transfer during cod rehydration. All parameters studied were influenced by the PEF pre‐treatment. Mass uptake $\left(∆M_{t}^{o}\right)$ increased significantly (*P* < 0.05) at the end of the process after both PEF treatments applied in the present study. Although the initial rate of mass uptake was significantly higher in the control sample (higher value of 1/*k*_1_), at the end of the process, the mass uptake at equilibrium was significantly higher in PEF treated samples (higher value of 1/*k*_2_). Considering only the water fraction, its uptake $\left(∆M_{t}^{w}\right)$ was faster in the untreated (Control) samples at the beginning, whereas the PEF‐treated samples had values of water uptake comparable to those of the untreated product at the end of the rehydration process (i.e. on day 6). The total solute variations $\left({∆M}_{t}^{S}\right)$ show a greater loss in the untreated samples, with both the initial and final mass transfer rates remaining significantly higher. To better understand the loss of NaCl during the desalting process, the salt content was monitored throughout the rehydration process. Salt loss accounted for about 35% of the total solute loss in the control and PEF (1) treated samples, whereas the loss in PEF (2) was approximately 37%. Figure 1 clearly shows that the samples in both selected PEF pre‐treatments had a lower salt loss than the control samples throughout the rehydration process. The initial rate of salt loss was significantly higher in the samples treated with PEF (1), whereas a higher rate of salt loss was observed in the control samples at the end of the process.

**Table 1:** Peleg's kinetic model of mass and water uptake and solutes and salt loss during rehydration of dry‐salted cod

	*k*_1_	SE	*k*_2_	SE	*r* ^2^	1/*k*_1_	1/*k*_2_
Mass uptake							
Control	112.939	14.311	2.121	0.183	0.776	0.009 a	0.471 b
PEF (1)	158.749	15.068	1.282	0.178	0.656	0.006 b	0.780 a
PEF (2)	160.175	13.840	1.287	0.163	0.714	0.006 b	0.777 a
Water uptake							
Control	21.416	2.489	1.704	0.523	0.841	0.047 a	0.587 a
PEF (1)	54.872	5.305	1.447	0.079	0.707	0.018 b	0.691 a
PEF (2)	57.505	4.481	1.392	0.065	0.804	0.017 b	0.718 a
Solute loss							
Control	20.994	1.314	3.966	0.423	0.955	0.048 a	0.252 a
PEF (1)	42.286	2.780	5.774	0.066	0.915	0.024 b	0.173 b
PEF (2)	59.695	3.646	5.254	0.074	0.925	0.017 b	0.190 b
Salt loss							
Control	209.338	18.746	10.049	0.322	0.901	0.005 b	0.100 a
PEF (1)	58.213	7.117	16.841	0.232	0.795	0.017 a	0.059 b
PEF (2)	321.131	33.178	12.873	0.544	0.785	0.003 b	0.078 b

Different lowercase letters correspond to significantly differences (*P* < 0.05) between samples for each mass transfer parameters.

The water activity (*a*_*w*_) of untreated and PEF pre‐treated samples during the rehydration process of dry‐salted cod is shown in Table 2. The initial *a*_*w*_ value of the cod samples was 0.752. As expected, there was a progressive increase in a_w_ values for all samples during the rehydration process, with significantly (*P* < 0.05) higher values found in the control samples in almost all the cases. At the end of the process, the control and PEF (1) sample had an *a*_*w*_ value of 0.997, whereas PEF (2) had a significantly (*P* < 0.05) lower value of 0.993.

**Table 2:** Water activity (*a*_*w*_) of untreated and PEF treated samples during rehydration of dry‐salted cod

	Control	PEF (1)	PEF (2)
Time (h)	Mean ± SD	Mean ± SD	Mean ± SD
0	0.752 ± 0.001 a	0.752 ± 0.001 a	0.752 ± 0.001 a
4	0.923 ± 0.004 a	0.937 ± 0.006 b	0.932 ± 0.005 c
6	0.935 ± 0.004 a	0.945 ± 0.005 b	0.946 ± 0.005 b
24	0.986 ± 0.003 a	0.971 ± 0.004 b	0.971 ± 0.005 b
48	0.994 ± 0.002 a	0.989 ± 0.002 b	0.993 ± 0.003 a
72	0.998 ± 0.001 a	0.992 ± 0.003 b	0.993 ± 0.003 b
96	0.997 ± 0.002 a	0.998 ± 0.001 a	0.998 ± 0.002 a
120	0.997 ± 0.002 a	0.998 ± 0.002 a	0.998 ± 0.002 a
144	0.997 ± 0.002 a	0.993 ± 0.003 b	0.997 ± 0.002 a

Different lowercase letters correspond to significantly differences (*P* < 0.05) between groups at the same selected time.

## DISCUSSION

The present study aimed to investigate the applicability of PEF technology for increasing the rate of desalting/rehydration of dry‐salted cod. Two different PEF pre‐treatments, namely PEF (1) and PEF (2), were selected to understand whether the increased permeabilisation of cell membranes by the electroporation process could lead to an increase of mass transfer phenomena characteristic of salted cod rehydration (i.e. water uptake and leaching of salt). The selection of the specific energy inputs used in this study was based on preliminary experiments to optimise key PEF parameters. As expected, throughout the rehydration period considered in the present study (6 days), weight and water content increased while salt content decreased. When examining the kinetics of mass transfer, some discrepancies between the untreated (control) and PEF‐treated samples were observed. Furthermore, the empirical Peleg model adequately described the kinetics of cod desalting/rehydration (the coefficients of determination, *r* ^2^, are given in Table 1), with the sole exception of the PEF (1) mass change $\left(∆M_{t}^{o}\right)$, which had the lowest *r* ^2^ (0.656). The application of PEF technology had a positive effect on weight gain during cod rehydration and showed increased total mass variation compared to the untreated sample, irrespective of the treatment‐specific energy input applied [i.e. PEF (1) and PEF (2) achieved similar values of mass variation after 144 h of rehydration]. Specifically, the higher weight of the pre‐treated product is a result of the lower loss of solids from the food matrix. Indeed, both pre‐treatments resulted in higher solute retention than the untreated sample and, in particular, lower leaching of NaCl. Nevertheless, the calculated salt content in both pre‐treated sample was found to be in the range of commercial rehydrated cod products, namely approximately 10–20 g kg^−1^ NaCl [10.0 ± 7.0 g kg^−1^ NaCl control; 20.0 ± 6.0 g kg^−1^ NaCl PEF (1); 10.5 ± 6.0 g kg^−1^ NaCl PEF (2)].^4^ However, sensory evaluation of PEF treated products should be considered to confirm that the processed food material is suitable for consumption. Although it is known that salt concentrations above certain level can positively influence the water holding capacity of the protein matrix, in the present study, the final water uptake [$∆M_{t}^{w}$, calculated following Eqn (2)] was similar in all sample groups considered. Based on our results, we can speculate that the significant differences in the rehydration behaviour of pre‐treated and untreated salted cod samples may be a result of the structural changes promoted by the application of high voltage pulses. From a chemical point of view, another hypothesis of the detected differences could be the induced orientation by PEF on polar molecules, such as NaCl and proteins, with a consequent possible changes in their interactions and related mass transfer phenomena.

As highlighted in the Introduction, to the best of our knowledge, this is the first study to show how PEF technology could affect desalting of food materials. Other non‐thermal technologies have already been investigated with respect to improving the desalting process of cod, such as the application of high‐intensity ultrasound, which improved both moisture and NaCl diffusivity^10^ Because rehydration of fish can be simplified as the process opposite to salting, the influence of PEF pre‐treatment has also been shown to affect the salting process of fish fillets. As reported previously,^17^ although PEF pre‐treatment of sea bass fillet could positively influence salt intake, its application could negatively affect some qualitative properties of the food matrix, such as protein and lipid oxidation stability. In view of these considerations, further research should focus on the influence of PEF pre‐treatment on physicochemical properties during rehydration of cod fish.

## CONCLUSIONS

This exploratory study demonstrated the applicability of PEF technology as a pre‐treatment of dry‐salted cod for improving rehydration kinetics. We were able to show that selected PEF pre‐treatments positively influence the weight gain of cod during the desalting/rehydration process. In addition, less pronounced salt loss was observed in the pre‐treated fish, reaching (at the end of the desalting process) salt levels comparable to the commercial product. These results could indicate a beneficial application of the technology in an industrial setting because its application could lead to a higher process yield (higher weight gain) and the possibility of reducing water renewal, such that less NaCl is lost in the wastewater, resulting in lower water losses.

## CONFLICT OF INTERESTS

The authors declare that they have no conflicts of interest.
